# Radiosensitization of Non-Small Cell Lung Cancer Cells by the Plk1 Inhibitor Volasertib Is Dependent on the p53 Status

**DOI:** 10.3390/cancers11121893

**Published:** 2019-11-28

**Authors:** Jolien Van den Bossche, Andreas Domen, Marc Peeters, Christophe Deben, Ines De Pauw, Julie Jacobs, Sven De Bruycker, Pol Specenier, Patrick Pauwels, Jan Baptist Vermorken, Filip Lardon, An Wouters

**Affiliations:** 1 Center for Oncological Research (CORE), University of Antwerp, 2610 Wilrijk, Belgium; jolien.vandenbossche@uantwerpen.be (J.V.d.B.); marc.peeters@uza.be (M.P.); christophe.deben@uantwerpen.be (C.D.); ines.depauw@uantwerpen.be (I.D.P.); Julie.Jacobs@uantwerpen.be (J.J.); Patrick.Pauwels@uza.be (P.P.); JanB.Vermorken@uza.be (J.B.V.); filip.lardon@uantwerpen.be (F.L.); an.wouters@uantwerpen.be (A.W.); 2 Department of Oncology, Antwerp University Hospital, 2650 Edegem, Belgium; pol.specenier@uza.be; 3 Department of Pathology, Antwerp University Hospital, 2650 Edegem, Belgium; 4 Molecular Imaging Center Antwerp (MICA), University of Antwerp, 2610 Wilrijk, Belgium; Sven.DeBruycker@uantwerpen.be

**Keywords:** polo-like kinase 1, targeted therapy, volasertib, p53, non-small cell lung cancer, radiotherapy, senescence

## Abstract

Polo-like kinase 1 (Plk1), a master regulator of mitotic cell division, is highly expressed in non-small cell lung cancer (NSCLC) making it an interesting drug target. We examined the in vitro therapeutic effects of volasertib, a Plk1 inhibitor, in combination with irradiation in a panel of NSCLC cell lines with different p53 backgrounds. Pretreatment with volasertib efficiently sensitized p53 wild type cells to irradiation. Flow cytometric analysis revealed that significantly more cells were arrested in the G_2_/M phase of the cell cycle after the combination therapy compared to either treatment alone (*p* < 0.005). No significant synergistic induction of apoptotic cell death was observed, but, importantly, significantly more senescent cells were detected when cells were pretreated with volasertib before irradiation compared to both monotherapies alone (*p* < 0.001), especially in cells with functional p53. Consequently, while most cells with functional p53 showed permanent growth arrest, more p53 knockdown/mutant cells could re-enter the cell cycle, resulting in colony formation and cell survival. Our findings assign functional p53 as a determining factor for the observed radiosensitizing effect of volasertib in combination with radiotherapy for the treatment of NSCLC.

## 1. Introduction

Targeting mitosis is a validated approach in cancer research, and agents that affect the mitotic spindle are well-established components of many oncotherapeutic regimes in the clinic. Drugs like taxanes and vinca-alkaloids hamper the dynamic activity of microtubules and have been proven to be successful in platinum-based treatment schedules for the treatment of non-small cell lung cancer (NSCLC) patients. However, serious adverse effects as a consequence of interactions with tubulin in non-dividing differentiated cells, such as neurons, remain the dose-limiting factor of these chemotherapeutic agents [1,2]. New approaches to inhibit mitosis therefore rather target cardinal regulatory proteins. 

One of the most promising targets in this research field is Polo-like kinase 1 (Plk1), a serine/threonine kinase that plays an essential role in mitotic progression, thereby regulating multiple processes including mitotic entry, centrosome maturation, bipolar spindle formation, kinetochore assembly, and cytokinesis [3,4]. Moreover, recent data implicated the involvement of Plk1 in the response to DNA damage by (i) activation, maintenance, and recovery of the G_2_/M checkpoint; and (ii) stimulation of the DNA repair pathway [5,6]. Upregulation of Plk1 has been reported in several tumor types, including NSCLC. In these studies, high Plk1 expression levels correlated with poor patient prognosis, corroborating its importance in tumor progression and its potential as a therapeutic target [7,8,9,10,11]. So far, several small molecule inhibitors of Plk1 have been developed and are currently being evaluated in clinical trials. Volasertib, at present the lead agent in the category of Plk1 inhibitors, has been shown to induce mitotic arrest and cell death with a high efficacy in vitro, and to inhibit tumor growth in xenograft models [4,11,12,13]. In clinical trials, volasertib has been well tolerated, and has shown encouraging antitumor activity in patients with acute myeloid leukemia (AML) [14,15]. However, the highly promising preclinical data obtained in solid tumor types, including NSCLC, could only be confirmed to a lesser extent in patients, with partial response reported in only a limited number of cases [4,11]. Nevertheless, the small proportion of patients with meaningful clinical benefit might be masked among a larger proportion of patients who fail to benefit from Plk1 inhibition since no meaningful biomarkers were taken into account in most early phase clinical trials involving NSCLC [11]. Moreover, a significant percentage of patients reached stable disease, leaving an interesting therapeutic window for improving response rates to volasertib in patients with a favorable biomarker profile for Plk1 inhibition, such as p53 [4,11]. 

NSCLC, accounting for an estimated 80–90% of all lung neoplasms, is the most lethal type of cancer worldwide, with a 5 year survival varying from 4–17%, depending on stage and regional differences [16,17]. Despite the introduction of numerous new treatment modalities, surgery, traditional chemotherapeutic agents and radiotherapy remain the backbone for the treatment of NSCLC. To date, the treatment of choice for early stage (stage I/II) patients is surgical resection, either or not combined with chemotherapy. Otherwise, inoperable stage I/II NSCLC patients receive stereotactic radiotherapy. For patients with locally advanced stage III NSCLC, platinum-based chemotherapy in combination with radiotherapy is the preferred treatment option next to surgery if resectable. Chemotherapy, consisting of platinum-based doublets, whether or not in combination with immunotherapy-dependent on histology, molecular pathology, age, performance status, and comorbidities-is the standard-of-care first line treatment for the majority of metastatic (stage IV) NSCLC patients [16,17,18,19]. As such, almost all NSCLC patients receive chemotherapeutic agents and/or radiotherapy during their treatment process. Nevertheless, these conventional treatment modalities have reached a plateau in treatment efficacy due to the occurrence of serious side effects, with median overall survival rates for advanced NSCLC patients of approximately eight months and overall response rates of 19–30% [20]. Therefore, it becomes increasingly important to address the combined effect of agents targeting cardinal regulatory proteins of mitosis, such as Plk1, with conventional treatment modalities, in order to further improve outcome of patients diagnosed with NSCLC.

Here, we investigated the combination of volasertib with radiotherapy in more depth. The rationale for combining Plk1 inhibition with radiotherapy is supported by our previous study on volasertib monotherapy in NSCLC cell lines differing in p53 background [21]. In this study, treatment with volasertib caused cells to accumulate in the G_2_/M phase, which is the most radiosensitive cell cycle phase [22]. As such, we postulate that pretreatment with volasertib holds great promise to increase radiosensitivity. However, data on the combination of volasertib with radiotherapy in NSCLC is sparse. Therefore, in the present study, we aimed to examine the in vitro therapeutic effects of volasertib combined with ionizing radiation in a panel of NSCLC cell lines with different p53 backgrounds.

## 2. Results

### 2.1. Volasertib Radiosensitizes p53 Wild Type Cells in a Concentration-Dependent Manner

**Figure 1:**
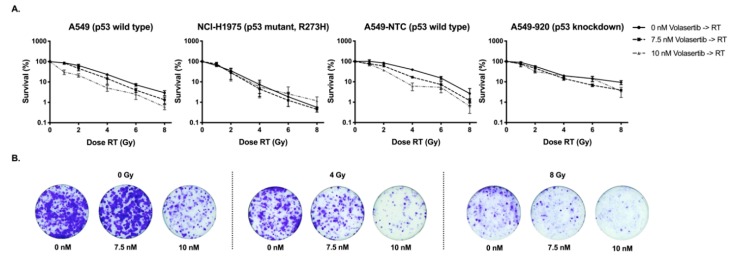
Clonogenic survival after pretreatment with volasertib (0–10 nM, 24 h), followed by irradiation (0–8 Gy) in A549, NCI-H1975, A549-NTC, and A549-920 cells: (**A**) Radiation dose-response curves after the combination treatment. Survival was determined by the clonogenic assay 10 days (d) after irradiation and corrected for the cytotoxic effect of volasertib monotherapy. Data points represent mean values from at least three experiments and are presented as mean ± standard deviation (SD); (**B**) Representative images of A549 cells after staining with crystal violet 10 d post-irradiation.

To investigate the potential interaction between volasertib and radiotherapy (RT), both p53 wild type (A549, A549-NTC) and p53 knockdown/mutant (A549-920, NCI-H1975) cells were exposed to volasertib (0–10 nM) for 24 h and followed by irradiation (0–8 Gy). As shown in Figure 1 and Table 1, a concentration-dependent radiosensitizing effect of volasertib was observed only in the p53 wild type cell lines. In both A549 and A549-NTC cells, the half-maximum inhibitory dose (ID_50_-value) for irradiation significantly decreased with increasing concentrations of volasertib (*p* < 0.001) (Table 1). This effect was the strongest in the A549 cell line, with a decrease in ID_50_-value from 2.64 ± 0.20 Gy for radiotherapy alone to 0.66 ± 0.07 Gy when 10 nM volasertib was added to the cells 24 h before irradiation. The observed radiosensitizing effect was further confirmed by calculating the dose enhancement factor (DEF), which ranged from 1.32 ± 0.12 to 4.07 ± 0.59 in A549 cells and from 1.56 ± 0.07 to 2.24 ± 0.21 in A549-NTC cells (Table 1). In contrast, 24 h treatment with volasertib before irradiation resulted in an additive effect in A549-920 and NCI-H1975 cells, with DEFs ranging from 1.44 ± 0.39 to 1.50 ± 0.07 and from 0.97 ± 0.26 to 1.02 ± 0.33, respectively. In these p53 knockdown/mutant cell lines, no significant differences were observed between the ID_50_-values of radiotherapy alone compared to the ID_50_-values of the combination regimen (*p* ≥ 0.085).

As such, volasertib could only enhance cellular sensitivity to irradiation in NSCLC cells with wild type p53.

### 2.2. Treatment with Volasertib Followed by Irradiation Induces Mitotic Arrest

**Figure 2:**
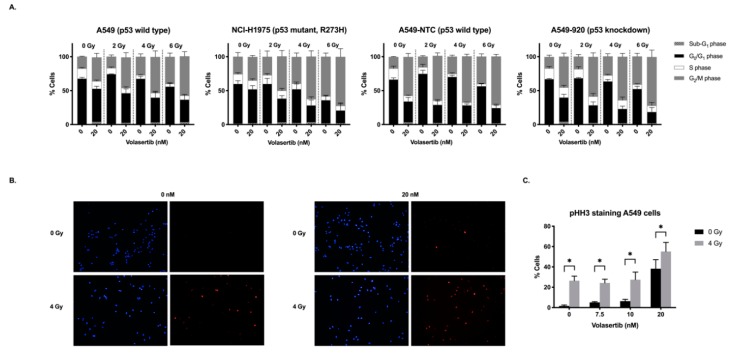
Cell cycle analysis 24 h after pretreatment with volasertib (0–20 nM, 24 h), immediately followed by irradiation (0–6 Gy). (**A**) The percentage of cells in each phase of the cell cycle is presented as mean ± SD of at least three experiments. Cells were stained with propidium iodide (PI) and DNA content was determined by flow cytometric analysis. Cells were divided in four groups; Sub-G_1_ phase (<2 N), G_0_/G_1_ phase (2 N), S phase (2 N–4 N), and G_2_/M phase (4 N). The percentages of the cells in each phase of the cell cycle for all treatment conditions are summarized in Table 2. (**B**) Immunofluorescence staining of pHH3 (red), a mitotic marker, in A549 cells 24 h after the combination treatment. Nuclei were stained with DAPI in blue (40×). (**C**) The percentage of positive pHH3 A549 cells 24 h after the combination treatment. * *p*-value < 0.050.

The effect of pretreatment with volasertib (0–20 nM) on radiation-induced cell cycle changes was examined in A549, A549-NTC, A549-920, and NCI-H1975 cells 24 h after irradiation (0–6 Gy) using flow cytometry. As presented in Figure 2A, treatment with either volasertib or irradiation (as of 2 Gy) as monotherapy resulted in a dose-dependent increase in the G_2_/M phase population, accompanied by a dose-dependent decrease in the percentage of G_0_/G_1_ phase cells. Pretreatment with volasertib 24 h before irradiation enhanced this effect. Table 2 summarizes the percentage of the cells in each phase of the cell cycle for all treatment conditions.

The effects of volasertib and irradiation as well as their interaction on the percentage of cells in each phase of the cell cycle were statistically analyzed using a linear regression model. A significant interaction indicates that the effect of irradiation on the cell cycle distribution is dependent on the concentration of volasertib and vice versa. In the absence of a significant interaction, effects of irradiation and volasertib work independent of each other, indicating an additive effect. In this case, the main effects of both monotherapies were calculated. With regard to the effect of the combination therapy on the percentage of G_2_/M phase cells, no significant interaction was observed between volasertib and radiotherapy, in all cell lines tested (*p* > 0.050). As expected, the main effects of either volasertib treatment or irradiation on the cell cycle distribution revealed a significant increase in the G_2_/M population (both *p* < 0.001). When both therapies were combined, an additive effect on the percentage of cells in the G_2_/M phase was seen. Indeed, compared to both monotherapies, significantly more cells were arrested in the G_2_/M phase when cells were pretreated with volasertib (20 nM) before irradiation (6 Gy), in three out of four cell lines (*p* ≤ 0.005). For example, in the A549 cell line, 17.48 ± 0.48% of the untreated cells were detected in the G_2_/M phase, with an increase to 35.10 ± 5.94% and 39.80 ± 5.53% after treatment with 20 nM volasertib or 6 Gy irradiation as monotherapy, respectively. Combination of these doses in the A549 cell line resulted in 57.93 ± 6.83% of the cells arrested in the G_2_/M phase.

To confirm these results, we performed immunofluorescent staining for phosphorylated histone H3 (pHH3), a mitotic marker, in the parental A549 cell line (Figure 2B). As shown in Figure 2C, for volasertib monotherapy, treatment with 20 nM volasertib resulted in a significant increase in the percentage of mitotic cells compared to untreated samples (*p* < 0.001). Likewise, irradiation with 4 Gy revealed a significant higher amount of pHH3-positive cells compared to 0 Gy (*p* < 0.001). In accordance with the flow cytometry data, the highest percentage of pHH3-positive cells was observed when A549 cells were pretreated with 20 nM volasertib followed by irradiation (4 Gy). Nevertheless, no significant interaction was found between the Plk1 inhibitor and radiotherapy (*p* = 0.668), indicating an additive effect on the mitotic arrest between both therapies. The mitotic arrest was accompanied by a significant decrease in the percentage of G_0_/G_1_ and S phase cells in all cell lines tested. In three out of four cell lines tested, the decrease in the percentage of cells in the G_0_/G_1_ phase was significantly higher in the combination group (20 nM volasertib, followed by 6 Gy) compared to both monotherapies (*p* ≤ 0.043).

Overall, volasertib and radiotherapy interacted in an additive manner with regard to the cell cycle distribution after the combination regimen, resulting in significantly more mitotic-arrested cells after pretreatment with volasertib followed by irradiation.

### 2.3. The Combination of Volasertib with Radiotherapy Does Not Result in Synergistic Induction of Apoptotic Cell Death

**Figure 3:**
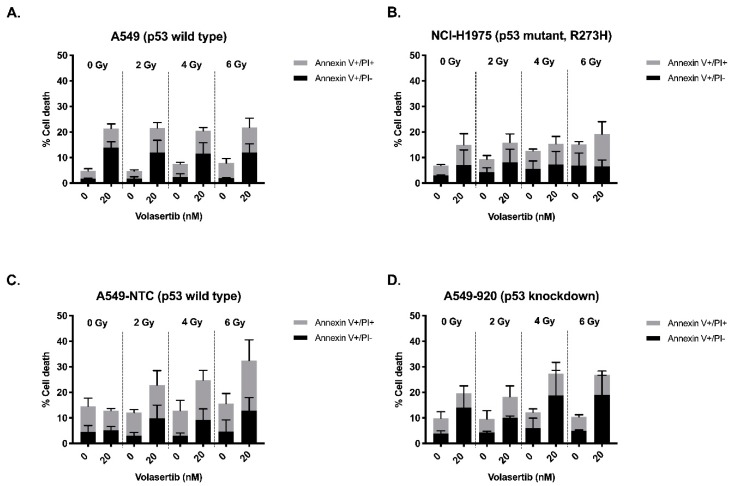
Apoptotic cell death 72 h after pretreatment with volasertib (0–20 nM, 24 h), followed by irradiation (0–6 Gy) in A549 (**A**), NCI-H1975 (**B**), A549-NTC (**C**), and A549-920 (**D**) cells. Cells were labeled with annexin V-FITC and PI and measured by flow cytometric analysis. Data is presented as the mean percentages of apoptotic cells (i.e., annexin V+/PI− and annexin V+/PI+) after the combination treatment; *n* > 3.

We assessed the induction of apoptotic cell death after treatment with volasertib (24 h, 0–20 nM) followed by irradiation (0–6 Gy), using the annexin V-FITC/PI and annexin V-APC/PI flow cytometric assays. As shown in Figure 3, in A549 and NCI-H1975 cells, volasertib monotherapy resulted in a dose-dependent increase in apoptotic (i.e., annexin V+/PI− and annexin V+/PI+) cells. Correspondingly, after incubation with volasertib, a decrease of viable (annexin V−/PI−) cells was observed from 94.01 ± 1.33% (0 nM) to 70.30 ± 7.71% (20 nM) and from 92.53 ± 0.80% (0 nM) to 83.37 ± 12.04% (20 nM), in the A549 and NCI-H1975 cell line, respectively. In A549-920 cells, treatment with volasertib induced an increase in the early apoptotic cell (Annexin V+/PI−) population as well as a decrease of viable cells from 89.20 ± 2.70% (0 nM) to 79.33 ± 8.57% (20 nM). In contrast, in the A549-NTC cell line, no effect of volasertib monotherapy could be observed on the percentage of apoptotic and viable cells (84.68 ± 5.37% (0 nM) versus 85.38 ± 1.95% (20 nM)). In the NCI-H1975 cell line, radiotherapy alone resulted in an increase of the percentage of apoptotic cells after irradiation with the highest dose (6 Gy). No effect of radiotherapy as monotherapy could be observed in the other cell lines. Except for A549-NTC cells, combination of volasertib and irradiation did not result in a higher percentage of apoptotic cells compared to volasertib or radiotherapy as monotherapy. The percentages of living and death cells for all treatment conditions are summarized in Table 3.

To statistically assess whether pretreatment with volasertib had an influence on induction of apoptotic cell death after radiotherapy, a linear regression model was fitted. In A549, NCI-H1975, and A549-920 cells, the interaction was found to be not significant for both viable and apoptotic cells (*p* ≥ 0.207). This indicates that the effect of radiation on the induction of apoptotic cell death was not dependent on the concentration of volasertib in these cell lines and vice versa. On the other hand, a significant interaction between volasertib and radiation was found for both viable and apoptotic cells in the A549-NTC cell line (*p* ≤ 0.031), indicating that the effect of volasertib is dependent on the radiation dose, and vice versa. For example, no significant increase in the induction of apoptosis was observed when the A549-NTC cell line was treated with volasertib or radiotherapy as monotherapy. However, pretreatment with 20 nM volasertib before 6 Gy irradiation induced a significant increase in apoptotic cells (*p* ≤ 0.034), accompanied by a significant decrease in the percentage of viable cells (*p* = 0.001), compared to untreated A549-NTC cells. For example, in untreated A549-NTC cells, 84.68 ± 5.37% of the cells were viable, in contrast to 63.34 ± 10.45% after treatment with 20 nM volasertib followed by 6 Gy irradiation.

In conclusion, the observed radiosensitizing effect of volasertib could not be explained by the induction of apoptotic cell death, except for the A549-NTC cell line.

### 2.4. Treatment with Volasertib Followed by Irradiation Induces Cellular Senescence, Especially in p53 Wild Type Cells

**Figure 4:**
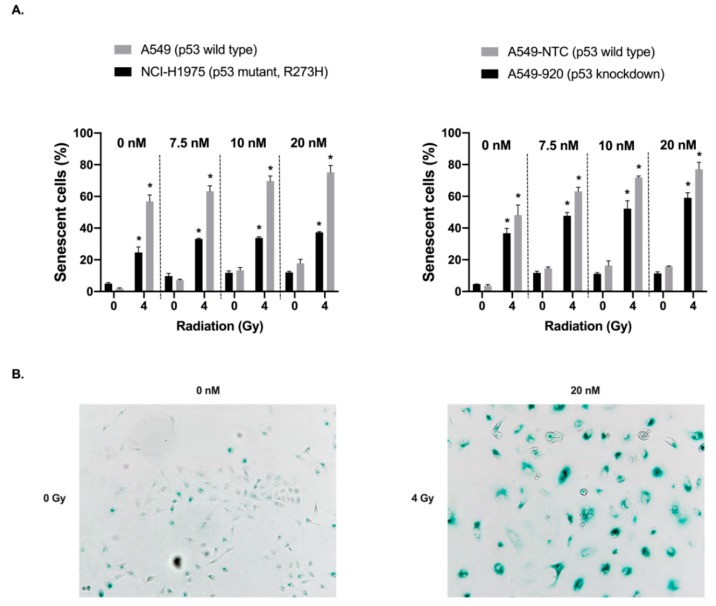
Induction of cellular senescence after the combination treatment of Plk1 inhibition and radiotherapy. (**A**) Percentage of β-galactosidase-positive cells after the combination treatment. Cells were treated with the Plk1 inhibitor (0–20 nM) for 24 h, followed by irradiation (0–4 Gy) and an incubation period of 72 h in drug-free medium. Data are presented as the mean percentages of β-galactosidase positive cells from at least three experiments. * *p*-value < 0.001 compared to non-irradiated sample. (**B**) X-gal staining of A549 cells 72 h after the combination treatment (20×).

Cellular senescence was investigated using a β-galactosidase staining kit in both p53 wild type and knockdown/mutant cells 72 h after exposure to volasertib (0–20 nM, 24 h) followed by irradiation (0–4 Gy). As shown in Figure 4, an increase in β-galactosidase staining was observed in all cell lines after treatment with volasertib and/or irradiation, which was further supported by the morphological readouts of cellular senescence.

We observed that both treatment with volasertib or irradiation alone resulted in a dose-dependent increase in the percentage of senescent cells. As a result, significantly more β-galactosidase-positive cells were detected when cells were pretreated with volasertib (20 nM) before irradiation (4 Gy) compared to both monotherapies (*p* < 0.001). Interestingly, when comparing p53 wild type A549 versus p53 mutant NCI-H1975 cells, a significant increase in the induction of cellular senescence was observed in both cell lines with increasing irradiation and volasertib doses. However, in the A549 cell line, the difference in the percentage of β-galactosidase positive cells between the irradiated and non-irradiated group was significantly larger compared with NCI-H1975 cells (*p* < 0.001). Similarly, when comparing the A549-NTC (p53 wild type) and A549-920 (p53 knockdown) cell lines, the effect of volasertib was larger in the p53 wild type A549-NTC cells within each irradiation dose compared to the A549-920 cells without functional p53, with a trend towards significance (*p* = 0.060). For an equal dose of volasertib, the percentages of senescent cells were consistently higher in the irradiated group compared to the non-irradiated group, and this difference was larger in the A549-NTC cell line (*p* < 0.001).

Statistical analysis using an ANCOVA model demonstrated an additive effect between volasertib treatment and irradiation in A549 (p53 wild type) and NCI-H1975 (*TP53* mutant) cells (*p* > 0.128) with regard to the induction of cellular senescence. As such, the effect of radiotherapy on the induction of cellular senescence is not influenced by the concentration of volasertib and vice versa. For the A549-NTC (p53 wild type) and A549-920 (p53 knockdown) cell lines, the interaction between volasertib and irradiation was found significant (*p* ≤ 0.005), indicating a more pronounced effect of volasertib dose in the irradiated group and thus a synergistic induction of senescence after the combination treatment. These results are also supported by the clonogenic assay (Figure 1) where most cells with functional p53 were permanently growth-arrested in contrast to p53 knockdown/mutant cells that were more feasible to re-enter cell cycle, resulting in colony formation and thus survival.

Overall, these results indicate increased cellular senescence after the combination therapy, especially in cells with functional p53. 

## 3. Discussion

Despite the discovery of several promising new treatment modalities in the last decade, surgery, traditional chemotherapeutics, and radiotherapy remain the backbone for the treatment of the majority of NSCLC patients [16,17,18,19]. Nevertheless, since NSCLC is still the most lethal cancer type worldwide, novel combination strategies are currently being investigated to improve the poor survival rates [16,17,20]. Elevated levels of Plk1, a crucial kinase during mitotic cell division, have been described in multiple cancer types, including NSCLC, with high expression levels being associated with poor survival [7,8,11]. Notwithstanding encouraging preclinical effectiveness of Plk1 inhibition, clinical trials with volasertib have shown only mild antitumor activity as a single agent [9,11]. As new combination strategies are key to improving clinical outcome of NSCLC patients, we aimed to study the in vitro effects of volasertib in combination with radiotherapy. We recently reported that NSCLC p53 wild type cell lines were more sensitive to volasertib monotherapy, suggesting, in line with other studies, that p53 might be a predictive biomarker for Plk1 inhibition in NSCLC [21,23,24]. However, no information is currently available on the Plk1-p53 axis in combination strategies for NSCLC. Therefore, we included a panel of isogenic NSCLC cell lines with different p53 backgrounds. 

Around 50% of all cancer patients and between 28% and 53% of the NSCLC patients receive radiotherapy either alone or in combination with other therapies during their treatment period [25,26]. Therefore, a lot of patients would benefit from any progress in the treatment with radiotherapy. Irradiation has been demonstrated to result in unrepaired DNA damage and subsequent mitotic catastrophe, further strengthening the hypothesis that radiotherapy combined with a mitotic inhibitor can improve treatment efficacy [27]. Taxanes (e.g., paclitaxel and docetaxel) have been proven to be valid radiosensitizers, however, severe side effects such as peripheral neurotoxicity remain the dose-limiting factor. Targeting the regulatory components of mitotic cell division, including Plk1, is expected to result in fewer side effects. Importantly, Lund–Andersen et al. proposed that the treatment schedule is critical for the radiosensitizing effect of Plk1 inhibition. Administration of a Plk1 inhibitor before irradiation can result in radiosensitization due to accumulation of cells in mitosis, which is the most radiosensitive cell cycle phase. In contrast, radiotherapy followed by Plk1 inhibition will prolong the G_2_ checkpoint arrest, leading to enhanced DNA repair and less cytotoxicity of the Plk1 inhibitor [22]. Hence, in the present study, NSCLC cell lines with different p53 backgrounds were treated with volasertib for 24 h followed by irradiation. Our results show that the Plk1 inhibitor acts as a radiosensitizer in p53 wild type cells but not in p53 knockdown/mutant cells. Interestingly, in line with findings from Noor et al. pretreatment with volasertib caused an accumulation of cells in the G_2_/M phase of the cell cycle in all cell lines tested, suggesting that factors other than the cell cycle phase also influence radiosensitivity. In accordance with our results, Yao et al. recently published an increase in radiosensitivity of human NSCLC cells, including A549, after pretreatment with volasertib. A more in-depth study of the molecular mechanisms revealed that the enhanced retention of γ-H2AX foci and the induction of mitotic catastrophe were related to a decrease in DNA damage repair efficacy in the combination treatment group, compared to irradiated cells without volasertib pretreatment. The unrepaired DNA damage in the combination treatment group relied on deficiencies in both the non-homologous end joining and homologous recombination repair pathways [25]. As such, the difference in radiosensitivity between p53 wild type and p53 knockdown/mutant NSCLC in our study can possibly be attributed to the role of p53 in the response to cellular stress, including DNA damage repair. 

In line with our findings, several other studies reported that the combination of Plk1 inhibition with radiotherapy leads to synergistic cell killing in vitro in multiple cancer types such as breast cancer, NSCLC, cervical epithelial adenocarcinoma, medulloblastoma, osteocarcinoma, glioblastoma, Merkel cell carcinoma, colorectal cancer, bladder carcinoma, oral cancer, and esophageal squamous cell carcinoma [8,28,29,30,31,32,33,34,35,36,37,38,39,40]. In vivo, Dong et al. demonstrated a synergistic inhibition of tumor growth and prolonged median survival in a tumor xenograft murine model with glioblastoma stem cells after treatment with volasertib and radiation [38]. However, Krause et al. were not able to demonstrate a direct cellular radiosensitization by volasertib in a head and neck squamous cell carcinoma xenograft model. Nevertheless, using a fractionated irradiation schedule with repeated Plk1 inhibition, a significant improvement in local tumor control was observed when compared to irradiation alone [41]. Using the small molecule Plk1 inhibitor TAK-960, Inoue et al. also demonstrated a significantly radiosensitizing effect in a NSCLC xenograft model (H1299) cells and in a cervical cancer xenograft model (HeLa cells) [37]. Therefore, combining Plk1 inhibition with radiotherapy remains an interesting therapeutic strategy.

In order to further unravel the difference in radiosensitization in p53 wild type versus p53 knockdown/mutant NSCLC cells, we investigated the cell cycle distribution, induction of apoptotic cell death and cellular senescence after volasertib followed by irradiation. Cell cycle distribution after irradiation with or without volasertib revealed that the combination therapy significantly decreased the G_0_/G_1_ population and increased the G_2_/M population compared to radiotherapy or volasertib monotherapy in all cell lines. Moreover, a significant increase in pHH3 staining in A549 cells was observed after the combination treatment, confirming a stronger mitotic block when cells are treated with volasertib before irradiation. These results are in accordance with the study of Chen et al., who also demonstrated an enhanced mitotic arrest after the combinatorial treatment in esophageal squamous cell carcinoma, as evidenced by flow cytometric analysis of the cell cycle distribution and cyclin B/pHH3 expression levels. In addition, they reported that pretreatment with volasertib resulted in an increase in cleaved poly (adenosine diphosphate-ribose) polymerase (PARP) after irradiation [27]. Nevertheless, in our study, only in the A549-NTC cell line, pretreatment with volasertib resulted in a significant increase in the percentage of apoptotic cells after irradiation compared to volasertib monotherapy. In the other three cell lines, no significant synergy on apoptotic cell death was observed when the combination treatment was compared with volasertib monotherapy. However, Inoue et al. pointed out the importance of the optimal time frame to induce the mitotic arrest, in addition to the mitotic arrest itself, in order to obtain the radiosensitizing effect of the Plk1 inhibitor TAK-960 [37].

Therapy-induced cellular senescence in cancer cells, or at least a senescence-like phenotype, has already been shown to be a crucial phenotype after chemotherapy [42,43,44,45,46], radiotherapy [44,45,47,48,49], and more recently also after Plk1 inhibition [21,50,51]. Cellular senescence is defined as a state of permanent cell cycle arrest in response to different damaging stimuli [45]. However, the phenotype associated with senescence is highly variable and heterogenous [46,52]. In line with our previous work [21], we showed that volasertib monotherapy induced significantly more senescence in p53 wild type cells compared to their p53 knockdown/mutant counterparts. These results are consistent with the studies of Kim et al. and Driscoll et al., who also demonstrated a role for p53 in the induction of cellular senescence after Plk1 inhibition [50,53]. Interestingly, combinatorial treatment of volasertib plus irradiation resulted in significantly increased cellular senescence compared to either therapy alone, with the highest percentage of cellular senescence in p53 wild type cells. As such, while most cells with functional p53 were permanently growth-arrested, more p53 knockdown/mutant cells were feasible to re-enter the cell cycle, resulting in colony formation and thus cell survival (Figure 1). These results are in line with other studies investigating the importance of p53 in the modulation of cellular senescence in cancer cells [43,44,46,54]. Nevertheless, a synergistic interaction between volasertib treatment and irradiation for the induction of cellular senescence could only be observed in the A549-NTC and A549-920 cell line, while an additive effect was observed in A549 and NCI-H1975 cells.

In conclusion, cellular senescence could be assigned as a possible explanatory factor for the observed differential radiosensitizing effect of the Plk1 inhibitor in p53 wild type versus p53 knockdown/mutant NSCLC cells. It is known that cancer cells that underwent cellular senescence remain viable and metabolically active via the senescence-associated secretory phenotype (SASP). Therefore, it is of great importance to further investigate the molecular pathways that are activated in senescent cells upon this combination treatment. In the context of the innovative and emerging two-step anticancer therapeutic concept, that first evokes senescence and then eliminates residual senescent cancer cells with senolytic agents [55,56], combining volasertib with radiotherapy to prime patients with functional p53 for senolytic agents could be a promising new treatment strategy to ultimately maximize therapeutic efficiency and patient outcome.

## 4. Materials and Methods

### 4.1. Cell Lines and Cell Culture Conditions

The NSCLC adenocarcinoma cell lines included in this study were the parental A549 cell line (CCL-185, p53 wild type, American type cell culture collection (ATTC), Rockville, MD, USA) and its isogenic derivatives A549-NTC (non-template control, functional p53) and A549-920 (p53 shRNA, p53 knockdown). In order to obtain these isogenic cell lines, A549 cells were transduced with a GIPZ lentiviral shRNA VGH5526-EG7157 viral particle set (Thermo Fisher Scientific, Whaltham, MA, USA), as described previously [57]. NCI-H1975 (CRL-5908, mutant non-functional p53, *TP53*^R273H^, ATTC, Rockville, MD, USA) cells were included as a *TP53* mutant cell line. A549 cells and its isogenic derivatives and NCI-H1975 were cultured in Dulbecco’s Modified Eagle Medium (DMEM (Gibco, Thermo Fisher Scientific, Whaltham, MA, USA)) and Roswell Park Memorial Institute (RPMI (Gibco, Thermo Fisher Scientific, Whaltham, MA, USA)) medium, respectively, each supplemented with 10% fetal bovine serum (FBS), 1% penicillin/streptomycin and 1% l-glutamine. Additionally, 1% sodium pyruvate was added to the RPMI medium. All cell culture reagents were purchased from Life Technologies (Ghent, Belgium). Cells were maintained as monolayers in exponential growth in a 5% CO_2_/95% N_2_ humidified incubator at 37 °C and confirmed free of mycoplasma contamination through regular testing (MycoAlert^®^ Mycoplasma Detection Kit, Lonza, Verviers, Belgium). For subsequent experiments, cells were harvested by trypsinization, automatically counted with a Scepter 2.0 device (Merck Millipore, Burlington, MA, USA) and plated as specified below.

### 4.2. Radiation Experiments

To analyze the radiosensitizing effect of volasertib, a clonogenic assay was performed [58,59]. Briefly, A549, A549-NTC, A549-920, and NCI-H1975 cells were plated in 6-well plates. Seeding densities varied from 1000 to 6200 cells per well, depending on the plating efficacy and planned radiation dose for each cell line. After 4 h incubation, allowing attachment to culture dishes, cells were exposed to volasertib (0–10 nM) (Selleck Chemicals, Huissen, The Netherlands) for 24 h. Next, cells were irradiated (0–8 Gy) at room temperature using an X-RAD 320 irradiation device (Precision X-ray Inc., North Branford, CT, USA). Following a 10 day incubation period, a time frame sufficient to form colonies of at least 50 cells, cells were fixed and stained with crystal violet and the area occupied by colonies was determined using Image J Software v.1.49 (open source software, Wayne Rasband, National Institutes of Health, Bethesda, MD, USA) [60]. Survival curves were generated after normalizing for the amount of volasertib- induced cytotoxicity. The ID_50_-values (i.e., radiation dose producing a survival fraction of 50%) were calculated using WinNonlin Software (Pharsight (Certara), Princeton, NJ, USA)). The radiosensitizing effect of volasertib was represented by the DEF using the following formula: DEF = (ID_50_ of irradiation alone)/(ID_50_ of irradiation plus volasertib). DEF = 1 suggests an additive radiation effect, DEF > 1, a supra-additive effect and DEF < 1 a sub-additive effect. 

### 4.3. Analysis of Cell Cycle Distribution

For cell cycle analysis, cells were seeded in 6-well plates and allowed to attach to culture dishes for 4 h. Volasertib (0–20 nM) was added to the culture media for 24 h and immediately followed by irradiation (0–8 Gy). Cell cycle analysis was performed 24 h after drug wash out using the CycleTEST^TM^ PLUS DNA reagent kit (Becton Dickinson, Franklin Lakes, NJ, USA), according to the manufacturer’s instructions. Samples were analyzed using a FACScan flow cytometer (Becton Dickinson, Franklin Lakes, NJ, USA), acquiring 10,000 events/sample. Histograms of DNA content were analyzed using FlowJo Software v.10.0.7 (Becton Dickinson, Franklin Lakes, NJ, USA) to determine the percentage of cells in each phase of the cell cycle. 

### 4.4. Analysis of Apoptotic Cell Death Induction

To investigate induction of apoptotic cell death, cells were plated in 6-well plates and after a recovery period of 4 h, cells were treated with volasertib (0–20 nM) for 24 h. Subsequently, cells were irradiated (0–8 Gy) and washed with drug-free medium. Seventy-two hours later, adherent plus floating cell fractions were collected and apoptotic cell death was evaluated using the annexin V-FITC apoptosis detection kit (Becton Dickinson, Franklin Lakes, NJ, USA), according to the manufacturer’s instructions. Flow cytometric analysis was performed on the FACScan flow cytometer (Becton Dickinson, Franklin Lakes, NJ, USA). Each sample was analyzed using 10,000 events/sample. Data were analyzed using FlowJo Software v.10.0.7 and presented as dot plots (annexin V plotted against PI staining). For A549-NTC and A549-920 cells, the number of apoptotic cells was determined using annexin V-APC due to the interference of FITC with the control protein turbo-GFP present in the vector. 

### 4.5. Analysis of Cellular Senescence

Cells were plated in 6-well plates 4 h before treatment with volasertib (0–20 nM) for 24 h. Immediately afterwards, cells were irradiated (0–8 Gy) and medium was refreshed by drug-free medium. 72 h later, cells were fixed and stained at pH 6.0 using a senescence β-galactosidase staining kit (Cell Signaling Technology, no. 9860, Leiden, The Netherlands). Plates were incubated with X-gal staining solution overnight at 37 °C in a dry incubator without CO_2_. Using a transmitted-light microscope (Olympus BX41 (Olympus Corporation, Shinjuku, Japan)), equipped with a Leica DFC450C camera (Leica, Wetzlar, Germany), blue staining was visualized in three random non-overlapping fields using 10× objective and 10× eyepiece for quantification. Percentage of senescent cells (morphology alterations combined with positive staining) was determined using Image J software v.1.49 (Becton Dickinson, Franklin Lakes, NJ, USA). 

### 4.6. Immunofluorescence Experiments

To determine whether the combination treatment of volasertib with irradiation enhanced mitotic arrest and/or DNA damage (dsDNA breaks), immunofluorescence experiments using the mouse monocloncal anti-pHH3 (Ser10) antibody (1:2000, Merck Millipore, no. 05-806, Burlington, MA, USA) and anti-γ-H2AX (Ser139) antibody (1:500, Merck Millipore, no. 05-636-AF488, Burlington, MA, USA), respectively, were performed. 72 h after irradiation, cells were fixed with ice-cold methanol, permeabilized with 0.1% Triton-X100/PBS and blocked with 1% BSA/PBS for 1 h. Next, cells were incubated overnight with the primary antibody at 4 °C, followed by 1 h incubation at room temperature with the secondary antibody, i.e., donkey anti-mouse IgG Alexa Fluor^®^ 555 conjugate (1:1000, Thermo Fisher Scientific, Whaltham, MA, USA). Slides were counterstained with DAPI and mounted. Images of sections stained with the anti-pHH3 antibody were taken using an Olympus BX51 standard research fluorescence microscope (Olympus Corporation, Shinjuku, Japan), equipped with an Olympus DP71 digital camera (Olympus Corporation, Shinjuku, Japan). Sections stained with the anti-γ-H2AX antibody were visualized with an Evos Cell Imaging System (Thermo Fisher Scientific, Whaltham, MA, USA). The percentage of positive pHH3 cells and the amount of γ-H2AX foci per cell were counted using Image J software v.1.49 (Becton Dickinson, Franklin Lakes, NJ, USA).

### 4.7. Statistics

All experiments were performed at least three times. Results are presented as mean ± standard deviation (SD). Statistical analyses were conducted using SPSS v.23 (SPSS Inc., Brussels, Belgium) and R v3.3.2 (Foundation for Statistical Computing, Vienna, Austria). *p*-values < 0.05 were considered significant. For the combination experiments with radiotherapy, a two-way ANOVA test was used to evaluate the impact of both the volasertib concentration and the cell line type on the ID_50_-value (radiotherapy) as dependent variable. A post hoc analysis with Tukey correction for multiple hypothesis testing revealed which groups differed significantly from another. The effect on the cell cycle distribution and apoptosis was performed for each cell line separately using a linear regression model with the percentage of cells in a specific phase of the cell cycle/cell death as dependent variable, and irradiation and concentration of volasertib as independent variables. If the *p*-value of the interaction between irradiation and volasertib was not significant, a model with the main effect was fitted. To test whether the effect of irradiation on the percentage of pHH3-positive cells was affected by volasertib, a two-way ANOVA test was conducted with the percentage of pHH3-positive cells as dependent variable. Again, a post hoc analysis with Tukey correction for multiple hypothesis testing was performed afterwards. Using an ANCOVA, we examined the effect of volasertib and irradiation on the induction of cellular senescence. Percentages of β-galactosidase-positive cells were entered as dependent variable, and volasertib and irradiation dose as independent variable. If the interaction *p*-value was not significant, the main effects of volasertib concentration and irradiation dose were investigated. Here, a post hoc analysis with Tukey correction for multiple testing was used to show differences in apoptotic cells between the four concentrations of volasertib tested.

## 5. Conclusions

The findings from our study both confirm and expand on previous preclinical studies, by demonstrating that targeting Plk1 with the small molecule inhibitor volasertib in combination with radiotherapy is a promising strategy for the treatment of NSCLC patients with functional p53. Cellular senescence could be assigned as a possible explanatory factor for the observed differential radiosensitizing effect of volasertib in p53 wild type versus p53 knockdown/mutant NSCLC cells. As such, combining volasertib with radiotherapy to prime patients with functional p53 for senolytic agents to eliminate residual senescent cancer cells is a promising new treatment strategy to ultimately maximize therapeutic efficiency and patient outcome. Such combinations hold great potential to achieve a shift from stable disease to partial or complete response rates in NSCLC patients with wild type p53. Additional studies will be required to investigate the therapeutic potential of this innovative combination regimen and to further optimize its safety, feasibility and clinical effectiveness.

## Figures and Tables

**Table 1:** ID_50_-values and DEFs for A549, A549-NTC, A549-920, and NCI-H1975 cells after pretreatment with volasertib (0–10 nM, 24 h), immediately followed by radiotherapy (0–8 Gy). Data are represented as mean ± SD of at least three experiments. DEF > 1 and DEF < 1 indicate radiosensitization and radioresistance, respectively.

A549	ID_50_	DEF
0 nM volasertib → RT	2.64 ± 0.20	/
7.5 nM volasertib → RT	2.02 ± 1.05	1.32 ± 0.12
10 nM volasertib → RT	0.66 ± 0.07	4.07 ± 0.59
**A549-NTC**	**ID_50_**	**DEF**
0 nM volasertib → RT	3.53 ± 0.28	/
7.5 nM volasertib → RT	2.26 ± 0.19	1.56 ± 0.07
10 nM volasertib → RT	1.59 ± 0.25	2.24 ± 0.21
**A549-920**	**ID_50_**	**DEF**
0 nM volasertib → RT	2.45 ± 0.19	/
7.5 nM volasertib → RT	1.80 ± 0.61	1.44 ± 0.39
10 nM volasertib → RT	1.63 ± 0.18	1.50 ± 0.07
**NCI-H1975**	**ID_50_**	**DEF**
0 nM volasertib → RT	1.43 ± 0.04	/
7.5 nM volasertib → RT	1.52 ± 0.37	0.97 ± 0.26
10 nM volasertib → RT	1.48 ± 0.43	1.02 ± 0.33

ID_50_-value: radiation dose producing a survival fraction of 50%; DEF: dose enhancement factor; SD: standard deviation; RT: radiotherapy.

**Table 2:** Percentage of A549, A549-NTC, A549-920, and NCI-H1975 cells in each phase of the cell cycle after pretreatment with volasertib (0–20 nM), followed by radiotherapy (0–6 Gy). PI staining was performed 24 h after irradiation. Data are represented as mean ± SD of at least three experiments.

Cell Percentages in Each Phase of the Cell Cycle										
A549	Condition	Sub-G_1_	G_0_/G_1_	S	G_2_/M	NCI-H1975	Sub-G_1_	G_0_/G_1_	S	G_2_/M
	0 nM volasertib → 0 Gy	0.09 ± 0.09	67.88 ± 1.71	14.78 ± 1.40	17.48 ± 0.48		1.42 ± 1.10	58.73 ± 4.46	14.30 ± 1.99	25.70 ± 5.98
	7.5 nM volasertib → 0 Gy	0.82 ± 1.21	64.90 ± 2.71	13.23 ± 1.75	20.73 ± 0.74		1.14 ± 0.62	57.83 ± 4.26	13.45 ± 2.11	27.48 ± 5.58
	12.5 nM volasertib → 0 Gy	2.65 ± 3.64	61.20 ± 3.35	12.73 ± 1.41	23.18 ± 1.50		2.02 ± 1.37	53.85 ± 3.16	12.64 ± 4.80	31.48 ± 5.62
	20 nM volasertib → 0 Gy	3.96 ± 1.02	49.13 ± 3.37	10.95 ± 1.54	35.10 ± 5.94		2.06 ± 1.25	50.18 ± 5.06	12.93 ± 2.15	34.63 ± 2.07
	0 nM volasertib → 2 Gy	1.12 ± 1.45	73.40 ± 0.22	8.95 ± 1.45	16.73 ± 2.34		1.61 ± 1.15	58.55 ± 8.01	12.87 ± 2.15	27.00 ± 8.37
	7.5 nM volasertib → 2 Gy	0.58 ± 0.37	64.93 ± 4.25	8.76 ± 1.61	25.88 ± 5.12		1.98 ± 1.50	54.70 ± 6.07	10.43 ± 1.66	33.03 ± 5.86
	12.5 nM volasertib → 2 Gy	2.10 ± 2.32	59.63 ± 8.45	8.03 ± 1.58	29.88 ± 7.97		2.12 ± 1.88	48.80 ± 2.69	10.32 ± 1.54	38.53 ± 5.33
	20 nM volasertib → 2 Gy	2.53 ± 0.88	44.00 ± 6.08	7.65 ± 1.33	44.97 ± 6.80		3.23 ± 1.61	35.33 ± 3.07	11.62 ± 3.15	50.50 ± 8.51
	0 nM volasertib → 4 Gy	0.39 ± 0.47	67.33 ± 3.39	5.33 ± 0.74	27.05 ± 4.10		2.37 ± 2.44	49.95 ± 7.47	8.24 ± 2.14	39.33 ± 5.70
	7.5 nM volasertib → 4 Gy	1.45 ± 0.95	53.78 ± 8.69	5.31 ± 0.73	39.58 ± 8.43		2.11 ± 0.89	39.23 ± 5.69	7.05 ± 2.83	51.15 ± 5.68
	12.5 nM volasertib → 4 Gy	1.22 ± 1.44	49.55 ± 9.11	5.06 ± 1.02	43.98 ± 8.01		2.27 ± 1.86	34.55 ± 5.12	7.67 ± 2.80	55.68 ± 5.98
	20 nM volasertib → 4 Gy	3.15 ± 0.26	36.77 ± 7.93	6.84 ± 2.43	53.03 ± 8.89		3.44 ± 1.15	25.00 ± 7.86	9.43 ± 2.95	62.48 ± 8.40
	0 nM volasertib → 6 Gy	0.29 ± 0.21	55.88 ± 5.54	3.94 ± 0.42	39.80 ± 5.53		1.83 ± 0.86	34.30 ± 6.00	5.76 ± 1.40	58.30 ± 5.26
	7.5 nM volasertib → 6 Gy	0.68 ± 0.69	45.13 ± 8.70	3.32 ± 0.23	50.83 ± 8.25		2.04 ± 0.96	25.15 ± 4.72	6.38 ± 2.56	66.20 ± 5.66
	12.5 nM volasertib → 6 Gy	1.08 ± 1.05	41.90 ± 7.49	4.44 ± 0.79	52.55 ± 6.91		1.52 ± 1.26	17.57 ± 2.02	5.34 ± 1.79	75.50 ± 5.11
	20 nM volasertib → 6 Gy	3.56 ± 1.02	33.40 ± 7.44	5.82 ± 1.51	57.93 ± 6.83		2.01 ± 0.88	18.95 ± 10.97	6.86 ± 2.87	72.10 ± 12.08
**A549-NTC**	**Condition**	**Sub-G_1_**	**G_0_/G_1_**	**S**	**G_2_/M**	**A549-920**	**Sub-G_1_**	**G_0_/G_1_**	**S**	**G_2_/M**
	0 nM volasertib → 0 Gy	0.27 ± 0.31	66.30 ± 2.26	16.30 ± 3.24	16.90 ± 0.62		0.45 ± 0.16	66.67 ± 0.83	15.83 ± 2.59	16.77 ± 2.55
	7.5 nM volasertib → 0 Gy	0.85 ± 0.38	61.03 ± 3.95	14.50 ± 3.80	23.20 ± 1.73		3.06 ± 1.17	57.20 ± 3.66	17.00 ± 3.46	22.57 ± 4.47
	12.5 nM volasertib → 0 Gy	1.37 ± 1.02	57.07 ± 3.74	13.57 ± 1.53	28.87 ± 2.71		2.43 ± 0.37	56.13 ± 2.12	17.07 ± 4.37	24.87 ± 6.37
	20 nM volasertib → 0 Gy	3.25 ± 1.60	30.97 ± 5.85	8.48 ± 0.73	57.00 ± 5.02		4.15 ± 2.40	35.93 ± 4.47	14.77 ± 3.01	45.37 ± 5.17
	0 nM volasertib → 2 Gy	0.24 ± 0.20	74.77 ± 5.05	9.69 ± 4.05	15.70 ± 1.13		0.47 ± 0.33	68.10 ± 0.79	13.37 ± 3.35	18.27 ± 3.04
	7.5 nM volasertib → 2 Gy	0.87 ± 0.46	62.97 ± 3.58	8.83 ± 2.66	27.70 ± 3.13		1.81 ± 0.66	53.23 ± 2.90	12.97 ± 4.06	32.30 ± 3.97
	12.5 nM volasertib → 2 Gy	1.37 ± 0.79	57.90 ± 3.40	8.16 ± 3.18	33.40 ± 3.11		2.09 ± 1.00	48.90 ± 2.75	11.00 ± 3.80	37.90 ± 7.04
	20 nM volasertib → 2 Gy	2.32 ± 2.28	26.90 ± 5.39	6.21 ± 1.37	65.93 ± 4.31		2.31 ± 0.81	26.30 ± 4.71	12.96 ± 4.27	58.27 ± 5.77
	0 nM volasertib → 4 Gy	0.72 ± 0.53	69.77 ± 2.14	4.44 ± 1.35	25.33 ± 1.97		0.54 ± 0.22	63.33 ± 2.53	8.76 ± 1.37	27.97 ± 3.71
	7.5 nM volasertib → 4 Gy	0.85 ± 0.55	54.83 ± 3.51	4.67 ± 0.57	39.87 ± 3.65		1.31 ± 0.57	43.70 ± 1.51	8.84 ± 2.03	46.10 ± 1.28
	12.5 nM volasertib → 4 Gy	0.77 ± 0.44	45.53 ± 8.47	4.46 ± 1.33	49.60 ± 9.61		1.87 ± 0.18	37.67 ± 2.49	10.36 ± 3.07	50.30 ± 2.59
	20 nM volasertib → 4 Gy	1.71 ± 1.21	26.80 ± 1.92	4.24 ± 0.40	67.87 ± 3.09		2.05 ± 0.84	21.17 ± 3.88	12.88 ± 4.43	63.97 ± 4.80
	0 nM volasertib → 6 Gy	0.40 ± 0.36	56.40 ± 3.16	3.29 ± 0.56	40.30 ± 3.73		0.62 ± 0.04	52.07 ± 3.74	6.59 ± 0.83	41.17 ± 4.63
	7.5 nM volasertib → 6 Gy	0.58 ± 0.67	43.17 ± 3.43	2.96 ± 0.27	53.37 ± 3.56		1.01 ± 0.54	33.80 ± 0.96	7.63 ± 1.53	57.83 ± 1.11
	12.5 nM volasertib → 6 Gy	0.82 ± 0.68	40.77 ± 3.43	3.30 ± 0.42	55.33 ± 3.75		1.21 ± 0.47	31.10 ± 4.34	7.72 ± 2.88	60.03 ± 6.72
	20 nM volasertib → 6 Gy	1.98 ± 0.70	22.57 ± 3.69	4.67 ± 0.73	71.20 ± 3.13		1.91 ± 1.34	16.80 ± 6.34	9.36 ± 4.49	71.60 ± 10.06

SD: standard deviation.

**Table 3:** Percentages of living and dead A549-A549-NTC, A549-920, and NCI-H1975 cells after pretreatment with volasertib (0–20 nM), followed by radiotherapy (0–6 Gy). Annexin V/PI staining was performed 72 h after irradiation. Data are represented as mean ± SD of at least three experiments.

Percentages of Living and Dead Cells										
A549	Condition	Annexin V−/PI−	Annexin V+/PI−	Annexin V+/PI+	Annexin V−/PI+	NCI−H1975	Annexin V−/PI−	Annexin V+/PI−	Annexin V+/PI+	Annexin V−/PI+
	0 nM volasertib → 0 Gy	94.01 ± 1.33	1.71 ± 0.30	3.16 ± 0.81	0.65 ± 0.21		92.53 ± 0.80	3.07 ± 0.17	3.87 ± 0.41	0.57 ± 0.38
	20 nM volasertib → 0 Gy	70.30 ± 7.71	13.97 ± 2.22	7.44 ± 1.76	1.61 ± 0.62		83.37 ± 12.04	7.12 ± 5.87	7.90 ± 4.33	1.60 ± 1.90
	0 nM volasertib → 2 Gy	93.10 ± 2.37	1.86 ± 0.66	2.88 ± 0.50	0.62 ± 0.11		89.87 ± 1.50	4.27 ± 1.69	5.07 ± 1.46	0.79 ± 0.44
	20 nM volasertib → 2 Gy	76.00 ± 7.45	11.97 ± 4.79	9.61 ± 2.11	2.46 ± 1.16		81.77 ± 10.48	8.13 ± 5.18	7.76 ± 3.36	2.35 ± 2.05
	0 nM volasertib → 4 Gy	93.06 ± 1.94	2.46 ± 1.27	5.13 ± 0.59	1.30 ± 0.10		86.33 ± 2.54	5.50 ± 3.20	7.13 ± 0.74	1.01 ± 0.36
	20 nM volasertib → 4 Gy	77.87 ± 5.44	11.6 ± 4.26	8.82 ± 1.31	1.76 ± 0.82		80.83 ± 9.55	7.32 ± 5.02	8.08 ± 2.89	3.78 ± 2.76
	0 nM volasertib → 6 Gy	90.96 ± 9.18	1.96 ± 0.16	5.90 ± 1.73	1.64 ± 0.27		83.57± 4.74	6.94 ± 4.84	8.29 ± 1.00	1.21 ± 0.89
	20 nM volasertib → 6 Gy	67.20 ± 9.18	11.94 ± 3.40	9.80 ± 3.65	1.49 ± 0.79		77.57 ± 09.33	6.56 ± 2.49	12.60 ± 4.88	2.68 ± 1.22
**A549-NTC**	**Condition**	**Annexin V−/PI−**	**Annexin V+/PI−**	**Annexin V+/PI+**	**Annexin V−/PI+**	**A549-920**	**Annexin V−/PI−**	**Annexin V+/PI−**	**Annexin V+/PI+**	**Annexin V−/PI+**
	0 nM volasertib → 0 Gy	84.68 ± 5.37	4.59 ± 2.45	9.94 ± 3.24	0.80 ± 0.19		89.20 ± 2.70	3.97 ± 0.99	5.90 ± 2.59	0.93 ± 0.46
	20 nM volasertib → 0 Gy	85.38 ± 1.95	5.24 ± 1.43	7.62 ± 0.79	1.79 ± 0.62		79.33 ± 8.57	14.03 ± 8.47	5.56 ± 3.01	1.08 ± 0.50
	0 nM volasertib → 2 Gy	86.96 ± 2.26	3.05 ± 1.29	9.04 ± 1.20	0.96 ± 0.15		89.73 ± 3.34	4.28 ± 0.53	5.28 ± 3.35	0.69 ± 0.18
	20 nM volasertib → 2 Gy	74.08 ± 10.20	9.94 ± 5.09	12.88 ± 5.69	3.12 ± 0.93		81.40 ± 0.95	10.16 ± 0.62	8.09 ± 4.27	0.98 ± 0.15
	0 nM volasertib → 4 Gy	84.72 ± 6.11	3.04 ± 1.09	9.83 ± 4.04	2.38 ± 1.28		87.15 ± 5.18	6.03 ± 3.89	6.16 ± 1.37	0.64 ± 0.19
	20 nM volasertib → 4 Gy	69.75 ± 6.38	9.15 ± 4.42	15.65 ± 3.78	5.46 ± 1.50		77.00 ± 2.78	18.85 ± 9.77	8.44 ± 4.43	1.22 ± 0.14
	0 nM volasertib → 6 Gy	82.50 ± 6.90	4.65 ± 4.52	10.96 ± 3.94	1.89 ± 0.74		88.73 ± 3.09	4.92 ± 0.37	5.49 ± 0.83	0.85 ± 0.36
	20 nM volasertib → 6 Gy	63.34 ± 10.45	12.88 ± 5.09	19.54 ± 8.13	4.23 ± 2.23		72.18 ± 8.98	19.00 ± 7.67	7.83 ± 1.53	0.99 ± 0.15

SD: standard deviation.
